# The Education, Screening, and Referral Practices of Pediatricians Surrounding Cerebral/Cortical Visual Impairment

**DOI:** 10.7759/cureus.113466

**Published:** 2026-07-27

**Authors:** John D Rogers, Harlan R Sayles, Richard H Legge

**Affiliations:** 1 Ophthalmology, University of Nebraska Medical Center, Omaha, USA; 2 Public Health, University of Nebraska Medical Center, Omaha, USA

**Keywords:** cerebral/cortical visual impairment, education, referral practices, survey, vision screening

## Abstract

Introduction

The timely recognition of pediatric cerebral/cortical visual impairment (CVI) often depends on appropriate screening and referral practices. The purpose of this study was to evaluate pediatricians' CVI education levels, visual dysfunction screening and referral practices, and potential use of hypothetical screening tools of varying lengths. The findings were intended to provide new insights into CVI screening among pediatricians and inform the development of more effective CVI screening tools.

Methods

A 60-question survey comprising multiple-choice, select-all-that-apply, Likert-type, and free-response questions was developed. The questions assessed pediatricians' general practice information, CVI education, visual dysfunction screening and referral practices, and the intended use of theoretical screening tools of varying lengths. The survey was distributed individually via email to all 396 pediatricians in Nebraska. Eighty-nine responses were analyzed.

Results

Our study revealed low levels of CVI education and awareness among respondents, along with limited visual dysfunction screening during many visits for children at risk for CVI. At the same time, many respondents expressed strong interest in additional CVI education if it were available. Respondents reported greater intended use of hypothetical screening tools requiring no more than two minutes to administer. The majority of respondents also reported being somewhat or highly likely to use a hypothetical mobile screening application described in the survey.

Conclusions

Increasing CVI education for pediatricians may be warranted, and developing brief, practical CVI screening tools may improve their acceptability among pediatricians. These results may provide a framework for the future development of such tools; however, future studies are needed to determine whether these approaches translate into increased clinical screening and earlier diagnosis of CVI.

## Introduction

Cerebral/cortical visual impairment (CVI) is the most common cause of childhood visual impairment in developed countries, yet it remains substantially underdiagnosed [1,2]. This prevents children with CVI from receiving proper education and care, further widening disparities in an already disadvantaged group [3,4]. Cerebral/cortical visual impairment results from an underlying disease process in the brain, rather than in the eye. In CVI, the brain cannot process visual information due to an underlying structural or functional defect [5]. Some children may have normal to near-normal visual acuity while others do not, and many children affected by CVI have comorbid neurological conditions [6], creating complicated clinical pictures with many varying presentations. As such, each child with CVI presents with a unique clinical picture that requires individualized assessment [7].

Pediatricians are often the first point of contact for identifying visual issues in children [5,8,9]; however, referral for and diagnosis of CVI are often delayed, with the average age at diagnosis being 4.6 years, suggesting missed opportunities for earlier identification [1]. Current guidelines recommend periodic visual assessments throughout childhood, with a focus on ocular disease and risk factors for amblyopia. However, specific recommendations for the screening and identification of CVI remain limited [10]. Although several CVI screening questionnaires exist, their validation has been limited, and screening practices remain largely unknown [11]. In addition, prior studies suggest that pediatricians may be uncomfortable performing vision screening, which may contribute to fewer referrals to ophthalmology [12]. Altogether, this suggests a potential lack of CVI screening among pediatricians, but no studies have specifically examined pediatricians' awareness of and clinical practice regarding CVI. In addition, because children with neurologic disease represent a population at particularly high risk for CVI, screening for visual dysfunction in this group may provide an opportunity for earlier recognition of children who warrant further evaluation for CVI.

Given this, we created a survey to primarily assess pediatricians' vision screening practices, particularly when seeing children with neurological disease. We also assessed pediatricians' CVI education, referral practices, and intended use of hypothetical screening tools. The purpose of this study was to provide new insights into opportunities for earlier identification of CVI among children with neurologic disease and to inform the development of more effective CVI screening tools.

## Materials and methods

Procedure

A 60-question survey consisting of multiple-choice, select-all-that-apply, Likert-type (where participants selected one of several ordered response options to indicate the degree of their agreement), and free-response questions was created. The creator of the survey was a board-certified ophthalmologist, with survey design input given by a survey methodologist at our institution. During the survey's creation, feedback and revisions were collaborative among these entities. This collaboration was intended to improve content and clarity, although the instrument was not formally validated prior to distribution. Survey questions were designed to focus on general practice information, education on CVI, visual dysfunction screening, and referral practices of pediatricians. The survey was distributed via REDCap [13,14], and all data were collected anonymously. Prior to completing the survey, respondents were informed of the purpose of the study, the risks and benefits, contact information, and that completion of the survey was voluntary. There were no incentives for completing the survey. This study was reviewed by the Institutional Review Board at our institution and determined to be exempt from full review (IRB #0723-24-EX). Informed consent was obtained via implied consent. Participants were instructed that completing the survey constituted consent to participate in the study. The survey was distributed individually via email to all 396 pediatricians in Nebraska. Responses were collected from March 2025 until December 2025. Reminder emails were sent approximately every two weeks. The complete survey instrument is provided in the Appendix. 

Participants

A complete list of pediatricians in Nebraska was obtained from the Nebraska Healthcare Workforce office. Licensed pediatricians who received the survey invitation and completed at least one survey question were eligible for inclusion in the study. At the time of the study, there were 396 pediatricians in the state of Nebraska, and 89 individuals who met the study criteria responded to the survey. Eleven respondents did not complete the entire survey, but their responses to the questions they answered were included in the analysis.

Measured variables of the survey

General Information

Participants were asked questions about the general characteristics of their practice, such as zip code, academic vs. non-academic, sub-specialty vs. general, and full-time vs. part-time. They were also asked about their years of practice.

Education on CVI

Participants were asked questions about their CVI education, including lectures or conferences they attended after their training. They were asked about their awareness of the CVI diagnosis process and their likelihood of attending future lectures/conferences on CVI.

Visual Dysfunction Screening

Participants were asked how often they screen children with neurologic disease for visual dysfunction. They were also asked a series of questions about their intended use of hypothetical screening tools of varying lengths, including whether the screening tool was accessible on a mobile device.

Referral Practices

Participants were asked about their referral practices for children with suspected CVI. They were then asked if the specialists they would refer these children to are within their geographic area and easily available for contact.

Statistical analysis

Study data were collected and managed using REDCap electronic data capture tools hosted at our home institution. Descriptive statistics were used to summarize survey responses via Microsoft Excel (Microsoft, Redmond, Washington). Responses are reported as frequencies and percentages, with percentages based on the number of responses to each question. Because the survey was anonymous, duplicate responses could not be definitively identified; however, responses were reviewed for obvious duplicate entries via zip code matching, and none were identified. Because individual survey questions were optional, respondents with incomplete surveys were retained, and available responses were analyzed on a question-by-question basis. Missing responses were excluded from analyses of the corresponding survey question. Questions 58-60 were included in the Appendix for completeness but were outside the scope of the present analysis and are not discussed in the results.

## Results

General information

Fifty-two (61.9%) respondents were general pediatricians, with an additional 20 (23.8%) as specialty pediatricians, four (4.8%) as neonatologists, four (4.8%) as pediatric intensivists, two (2.4%) as pediatric neurologists, and one (1.2%) as a pediatric hospitalist. One respondent listed both a general pediatrician and a neonatologist, and this figure is not included in the percentages above. Forty (48.2%) respondents have worked for more than 20 years, and 23 (27.7%) have worked for less than 10 years. Only two (2.4%) respondents have worked two years or fewer. Sixty-five (78.3%) respondents reported working full-time, with 38 (45.8%) working at an academic center. Of those who work in non-academic environments, 34 (75.6%) were in a large metropolitan area.

Education on CVI

**Figure 1:**
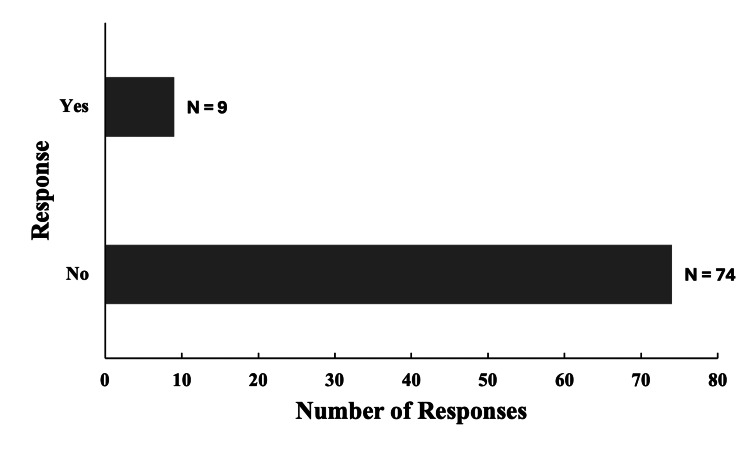
Reported attendance at CVI lectures or conferences (N = 83) The question generating this data was as follows: During your residency or fellowship, did you ever attend a lecture or conference on CVI? CVI - cerebral/cortical visual impairment

**Figure 2:**
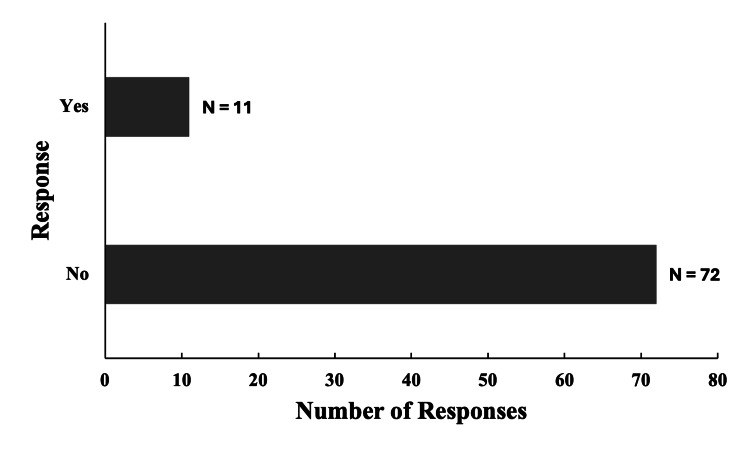
Reported continuing education lectures on CVI (N = 83) The question generating this data was as follows: In your continuing medical education experience since leaving training, have you ever attended a lecture on CVI? CVI - cerebral/cortical visual impairment

Seventy-four (89.2%) respondents did not attend a lecture or conference on CVI during their residency or fellowship, and 72 (86.7%) have not attended a lecture on CVI during their continuing medical education experience (Figure 1, Figure 2). Sixty-four (77.1%) respondents were somewhat likely or very likely to attend continuing medical education courses available at local or national professional meetings within two years. Of those who stated they were somewhat unlikely or very unlikely to attend continuing medical education courses, 12 (63.2%) listed lack of time as the reason. Additionally, 76 (96.2%) respondents were unaware of the criteria used to diagnose CVI, with 77 (97.5%) reporting that they are not able to make the diagnosis of CVI.

Regarding their general awareness of CVI, 26 respondents (33.3%) had not heard the term "CVI", and 22 (28.2%) had not heard the term "cortical vision impairment". Forty-nine (62.8%) had not heard the term "cerebral vision impairment", and 52 (66.7%) had not heard the term "brain-based vision impairment". Forty-three (55.1%) respondents did not know what CVI stands for. Furthermore, 77 (98.7%) had not heard of the Pediatric Cortical Visual Impairment Society.

Visual dysfunction screening

**Figure 3:**
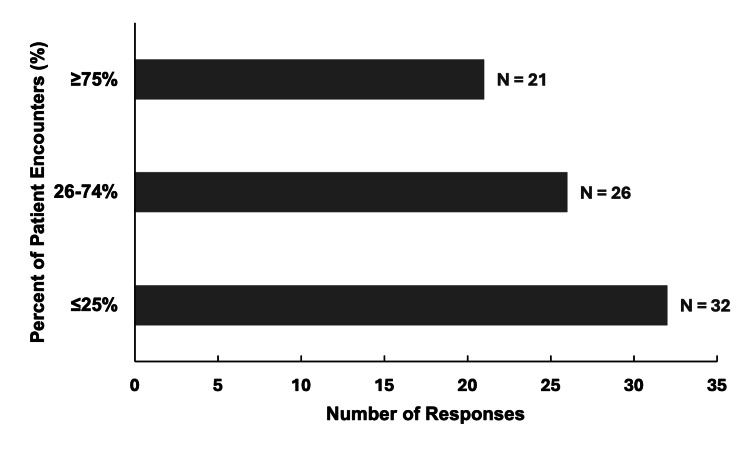
Frequency of visual dysfunction screening during patient encounters (N = 79)

Regarding reported screening practices for visual dysfunction, 53 (67.1%) respondents reported screening for visual dysfunction in children with neurological disease. Of those who screen for visual dysfunction, 45 (85.0%) reported doing so by questioning parents, and 47 (88.7%) reported doing so through physical exam. Nine (17.0%) reported using vision screening tools (e.g., Plus Optix, Welch-Allyn Vision Screener). The reported percentages of patient encounters in which this screening occurs are listed in Figure 3.

When asked about the intended use of hypothetical screening tools, 47 (59.5%) respondents reported they would use it at least 75% of the time if it consisted of three or fewer questions that took two minutes or less to administer. Fifty-one (64.6%) reported that they would use a screening tool if it consisted of two or fewer questions, involved one physical exam, and took two minutes or less to administer. Responses decreased to 21 (26.6%) if the screening tool consisted of five or fewer questions and took four minutes or less to administer, and they decreased further to nine (11.4%) if it consisted of 10 or fewer questions that took eight minutes or less to administer. Furthermore, 69 (87.3%) respondents reported that they would be somewhat or highly likely to use this tool if it were available as a free app on a mobile device and took five minutes or less to administer.

Referral practices

The most common specialty that respondents would refer children to after a positive theoretical or actual visual dysfunction screening test result was pediatric ophthalmology, followed by pediatric neuro-ophthalmology, pediatric neurology, ophthalmology, and neuro-ophthalmology. These and other accessibility results are shown in Table 1.

**Table 1:** Pediatrician referral practices and specialist availability

Specialist receiving referral	Number of pediatricians referring to specialist	Number reporting that the referred specialist is in their geographic area	Number reporting that the specialist is easily contacted if in geographic area
Neurologist	9 (11.4%)	7 (77.8%)	7 (100.0%)
Neuro-ophthalmologist	33 (41.8%)	25 (75.8%)	23 (92.0%)
Neuro-optometrist	5 (6.3%)	2 (40.0%)	2 (100.0%)
Occupational therapist	8 (10.1%)	8 (100.0%)	7 (87.5%)
Ophthalmologist	34 (43.0%)	34 (100.0%)	34 (100.0%)
Optometrist	5 (6.3%)	5 (100.0%)	5 (100.0%)
Pediatric Neurologist	36 (45.6%)	35 (97.2%)	35 (100.0%)
Pediatric neuro-ophthalmologist	42 (53.2%)	26 (61.9%)	26 (100.0%)
Pediatric ophthalmologist	53 (67.1%)	46 (87.8%)	46 (100.0%)
Pediatric optometrist	9 (11.4%)	7 (77.8%)	7 (100.0%)
Physiatrist	2 (2.5%)	2 (100.0%)	2 (100.0%)
Physical therapist	4 (5.1%)	4 (100.0%)	4 (100.0%)
Vision teacher	8 (10.1%)	7 (87.5%)	5 (71.4%)
None	1 (1.3%)	NA	NA

## Discussion

This study was conducted to understand CVI education, visual dysfunction screening, and referral practices among pediatricians. Our study found low levels of CVI education and awareness among survey respondents and limited reported screening for visual dysfunction in children with neurologic disease. Also, respondents reported greater intended use of hypothetical screening tools requiring two minutes or less to administer than of longer hypothetical tools, and the majority reported being somewhat or highly likely to use a hypothetical mobile screening application described in the survey.

Our data indicated a low level of CVI education and awareness among respondents. Even though most respondents were general pediatricians with over 20 years of experience, 43 (55.1%) did not know what CVI stands for. In addition, very few knew additional names for cortical visual impairment, such as "cerebral vision impairment" and "brain-based vision impairment". However, it must be acknowledged that these terms are not the standard terms typically used and may have influenced respondents' reported familiarity. Notably, 76 (96.2%) were unaware of the criteria used to diagnose CVI. This lack of awareness may be due to limited education, as seen in Figure 1 and Figure 2. Prior studies have highlighted low levels of CVI education even among optometrists and ophthalmologists [15]; thus, it is not surprising that these respondents experience the same.

Regarding screening rates, it is likely that low levels of education in CVI may be contributing to respondents' limited reported screening for visual dysfunction in children with neurologic disease. As seen in Figure 3, 32 (40.5%) respondents screen for visual dysfunction in children with neurological disease less than 25% of the time. As such, increasing CVI education for pediatricians, either during training or through continuing medical education, may help combat this discrepancy, especially since we observed that 64 (77.1%) were somewhat likely or very likely to attend continuing medical education courses at local or national professional meetings within the next two years. Overall, these data suggest that low levels of CVI awareness may mean that these respondents are seeing children with CVI but not recognizing it, possibly contributing to the underdiagnosis of CVI [9]. Of note, although the survey assessed screening for visual dysfunction rather than screening specifically for CVI, children with neurologic disease represent a high-risk population for CVI. Therefore, visual dysfunction screening in this population may represent an important opportunity for earlier recognition of children who warrant further evaluation for CVI.

As mentioned previously, tools to screen for CVI may be valuable in increasing detection rates, but time is often a limitation in pediatricians' screening practices [16]. Thus, any tool created to aid in CVI screening must be quick, and our data supported this. Regarding the development of a screening tool, most respondents reported they would use a two-minute screening tool during at least 75% of visits with children with neurologic disease. This number decreased substantially if the screening tool took four minutes or less to administer, and it decreased further if it took eight minutes or less. Thus, it appears that any screening tool developed must take two minutes or less to ensure that the majority use it. In addition, 69 (87.3%) reported that it was somewhat likely or highly likely they would use this tool if it were a free app on a cell phone or tablet and took five minutes or less to administer; however, the independent effect of mobile-device availability cannot be determined since this scenario differed from other scenarios in multiple ways. Overall, this suggests that any screening tool should take two minutes or less to administer to achieve greater usage, but future studies are needed to assess the independent effect of mobile-device availability.

Regarding referral practices shown in Table 1, respondents were similarly likely to refer to a neuro-ophthalmologist, an ophthalmologist, a pediatric neurologist, a pediatric neuro-ophthalmologist, or a pediatric ophthalmologist. Importantly, only five (6.3%) respondents indicated they would refer to a neuro-optometrist, even though neuro-optometrists can often diagnose CVI. This data may reflect the multidisciplinary nature of CVI evaluation, which often involves both ophthalmic and neurologic expertise. However, the absence of a clearly preferred referral destination may also suggest uncertainty regarding optimal referral pathways. Further efforts to establish clear referral practices for children with suspected CVI may facilitate more timely diagnosis, which may be accomplished by increasing CVI education as mentioned above. Otherwise, respondents generally reported knowing specialists within their geographic area and being able to obtain their contact information; however, survey questions did not assess appointment availability, wait times, insurance acceptance, or other barriers to accessing specialty care. Future studies are needed to determine whether barriers to these specialty providers exist.

In addition to our findings, this study has limitations that must be addressed. Because participation was voluntary, respondents may have had greater interest in or familiarity with CVI than nonrespondents, introducing the potential for response bias. Also, this data was gathered solely from Nebraska and may not represent the experiences of pediatricians in other geographic regions. Sample size is another limitation, as our response rate was 89/396, or approximately 22.5%. This modest number may limit the representativeness of our findings. Furthermore, this survey focused on self-reported vision screening and referral practices, which may differ from actual practices. Questions regarding a screening tool were hypothetical and may not accurately reflect future behavior if such a screening tool existed. Finally, although the survey was developed with expert input, it was not formally validated prior to distribution.

## Conclusions

Overall, this survey study assessed the CVI education, visual dysfunction screening, and referral practices among pediatricians in Nebraska. We found low levels of CVI education and awareness among respondents, and our study showed limited visual dysfunction screening in children with neurologic disease who are at risk for CVI. Our results suggest that increased continuing education in CVI for these physicians may alleviate some of these concerns. Regarding a screening tool, our findings indicate that a short screening tool (two minutes or less to administer) may lead to greater intended use, and respondents reported a high likelihood of using a mobile screening application as described in the survey. We hope these results provide a framework for the future development of such tools. Future studies are needed to determine whether these approaches translate to increased clinical screening and earlier diagnosis of CVI.

## Appendices

Survey questions distributed

Question 1: Do you see children with neurologic disease?

A.       Yes

B.       No

Question 2: Your primary office zip code:

(Free responses were collected)

Question 3: Which of the following best describes your practice? Choose one.

A.      General Pediatrician

B.      Neonatologist

C.      Pediatric Intensivist

D.      Specialty Pediatrician

E.       Pediatric Neurologist

F.       Other, Please Specify (Free responses collected as necessary)

Question 4: How many years have you been in practice since completing your last year of post-graduate training, such as your residency or fellowship? Choose one.

A.       0-2

B.       3-10

C.      11-20

D.      >20

Question 5: Do you consider yourself a full-time or part-time practitioner when you consider your time taking care of patients? Choose one.

A.       Full-time

B.       Part-time

Question 6: Would you describe your practice as primarily academic or non-academic? Choose one.

A.       Academic

B.       Non-academic

Question 7: If academic, which institution is your primary affiliation? Choose one.

A.       Boys Town National Research Hospital/Creighton University

B.       Children's/UNMC

C.       Nebraska Medicine/UNMC

D.       Other, Please list (Free responses collected as necessary)

E.        None

*If “Non-academic” was selected, respondents were directed to question 8.

Question 8: If non-academic, where is the majority of your practice time spent?

A.       Omaha metropolitan area or contiguous suburbs such as Bellevue

B.       Lincoln metropolitan area

C.      Within a 1-hour drive of the city limits of either the Omaha or Lincoln metropolitan areas

D.      Greater than a 1-hour drive from the Omaha or Lincoln metropolitan areas

Question 9: During your residency or fellowship, did you ever attend a lecture or conference on the topic of CVI?

A.       Yes

B.       No

Question 10: In your continuing medical education experience since leaving training, have you ever attended a lecture on CVI?

A.       Yes

B.       No

Question 11: If there were continuing medical education courses available at your local or national professional meetings, either live or virtual, how likely or unlikely is it that you would attend at least one in a 2-year period?

A.      Very likely

B.      Somewhat likely

C.      Somewhat unlikely

D.      Very unlikely

*If “Somewhat unlikely” or “Very unlikely” was selected, respondents were directed to question 12.

Question 12: If you answered "Somewhat unlikely" or "Very unlikely", why would that be? Choose all that apply.

A.      CVI is a low priority subject

B.      Lack of Time

C.      CVI is too complex

D.      CVI is too obscure

E.      Other

Question 13: Are you aware of diagnostic criteria that are used to diagnose CVI?

A.       Yes

B.       No

Question 14: Are you able to make the diagnosis of CVI?

A.       Yes

B.       No

Question 15: In children with neurologic disease, do you screen for visual dysfunction by any method?

A.       Yes

B.       No

*If “yes” was selected, respondents were directed to Question 16.

Question 16: If you answered "Yes", which method do you use to screen for CVI?  Check all that apply.

A.       Questioning Parents

B.       Physical Exam

C.      Other, Please describe (Free responses collected as necessary)

Question 17: If you screen for visual dysfunction in children with neurologic disease, please estimate the percentage out of all your patient encounters where you would do such screening. Choose one.

A.       Never

B.       About 1-10% of the time

C.       About 11-25% of the time

D.       About 26-74% of the time

E.       About 75-89% of the time

F.       About 90-100% of the time

Question 18: If you had a screening tool for visual dysfunction in children with neurologic disease consisting of no more than 3 questions that would take no more than 2 minutes to administer, please estimate how often you would use it. Choose one.

A.      Never

B.      About 1-10% of the time

C.      About 11-25% of the time

D.      About 26-74% of the time

E.       About 75-89% of the time

F.       About 90-100% of the time

Question 19: If you had a screening tool for visual dysfunction in children with neurologic disease consisting of no more than 2 questions and one physical exam test, that would take no more than 2 minutes to administer, please estimate how often you would use it. Choose one.

A.       Never

B.      About 1-10% of the time

C.      About 11-25% of the time

D.      About 26-74% of the time

E.       About 75-89% of the time

F.       About 90-100% of the time

Question 20: If you had a screening tool for visual dysfunction in children with neurologic disease consisting of no more than 5 questions that would take no more than 4 minutes to administer, please estimate how often you would use it. Choose one.

A.       Never

B.       About 1-10% of the time

C.       About 11-25% of the time

D.       About 26-74% of the time

E.       About 75-89% of the time

F.       About 90-100% of the time

Question 21: If you had a screening tool for visual dysfunction in children with neurologic disease consisting of no more than 10 questions that would take no more than 8 minutes to administer, please estimate how often you would use it. Choose one.

A.        Never

B.        About 1-10% of the time

C.       About 11-25% of the time

D.       About 26-74% of the time

E.       About 75-89% of the time

F.       About 90-100% of the time

Question 22: If there was a highly sensitive electronic screening tool for the detection of CVI using a free App on a cell phone or tablet device, and it would take no more than 5 minutes to administer, how likely would you be to use it?

A.       Highly likely

B.       Somewhat likely

C.       Not likely

Question 23: If you were using a screening tool of any kind, either hypothetically or actually, and the child would test "positive", would you refer the patient to any of the following specialists? Choose all that apply.

A.       None

B.       Neurologist

C.      Neuro-Ophthalmologist

D.      Neuro-Optometrist

E.       Occupational Therapist

F.       Ophthalmologist

G.      Optometrist

H.      Pediatric Neurologist

I.        Pediatric Neuro-Ophthalmologist

J.       Pediatric Ophthalmologist

K.       Pediatric Optometrist

L.       Physiatrist

M.     Physical Therapist

N.      Vision Teacher

O.      Other, Please specify

Questions 24-51 concerned the respondents’ answers to question 23. For each respective answer choice, the following question was asked:

For selection "[Answer from Question 23]", are such practitioners in your typical geographic referral area?

A.       Yes

B.       No

*If “yes” was selected for the above question, the following question was asked:

Can you easily obtain this practitioner’s name and contact information?

A.       Yes

B.       No

Question 52: Prior to this survey, have you heard the term "CVI"?

A.       Yes

B.       No

Question 53: Have you heard the term "Cortical Vision Impairment", other than when it was used in this survey?

A.       Yes

B.       No

Question 54: Have you heard the term "Cerebral Vision Impairment", other than when it was used in this survey?

A.       Yes

B.       No

Question 55: Have you heard the term "Brain-based Vision Impairment", other than when it was used in this survey?

A.       Yes

B.       No

Question 56: Prior to this survey, did you know what "CVI" stands for?

A.       Yes

B.       No

Question 57: Have you heard of the Pediatric Cortical Visual Impairment Society, www.pcvis.vision?

A.       Yes

B.       No

Question 58: If you have heard of the Pediatric Cortical Visual Impairment Society, did you know that you can refer patients to its website for information on CVI?

A.       Yes

B.       No

Question 59: Have you ever referred a patient to the Pediatric Cortical Visual Impairment Society website?

A.       Yes

B.       No

Question 60: Screening for vision impairment can be done with a child by having the child make eye contact with you at 1-3 feet of distance, then slowly move your head to see if he/she maintains eye contact. What is the earliest age you would feel comfortable conducting this vision screening technique?

A.       6 months

B.       1 year

C.      18 months

D.      2 years

E.       I don't think I could make this assessment.
